# A Noninvasive Gas Exchange Method to Test and Model Photosynthetic Proficiency and Growth Rates of In Vitro Plant Cultures: Preliminary Implication for *Cannabis sativa* L.

**DOI:** 10.3390/biology11050729

**Published:** 2022-05-10

**Authors:** Marco Pepe, Evangelos D. Leonardos, Telesphore R. J. G. Marie, Sean T. Kyne, Mohsen Hesami, Andrew Maxwell Phineas Jones, Bernard Grodzinski

**Affiliations:** Department of Plant Agriculture, University of Guelph, Guelph, ON N1G 2W1, Canada; pepem@uoguelph.ca (M.P.); dleonard@uoguelph.ca (E.D.L.); mariet@uoguelph.ca (T.R.J.G.M.); skyne@uoguelph.ca (S.T.K.); mhesami@uoguelph.ca (M.H.); bgrodzin@uoguelph.ca (B.G.)

**Keywords:** photosynthesis, in vitro culture, micropropagation, cannabis, plant tissue culture, gas exchange, LED, carbon metabolism, plant response

## Abstract

**Simple Summary:**

The gas exchange system presented herein integrates open-flow/force ventilation, LED technology, and micropropagation to determine the impact of environmental factors (e.g., [CO_2_], sucrose, light intensity) on the photosynthetic capacity of cultured plantlets. This system was developed and tested on *Cannabis sativa* L., an emerging crop of high economic value, for which micropropagation has become an important aspect of production. Since conventional micropropagation avenues can minimize photosynthetic performance, this system offers fresh opportunities to examine the role of light signaling and photosynthesis in micropropagation to investigate and overcome in-vitro-associated morphophysiological disorders. By maintaining [CO_2_] at controlled levels (400 and 1200 ppm) with calibrated light intensities, photosynthetic light response curves were prepared based on net carbon exchange rates (NCERs) to paint a picture of the dynamic, combinational influences of irradiance, [CO_2_], and additional factors on photosynthetic performance. Additionally, NCERs were continuously monitored during a 24 h light/dark period under standard conditions to provide estimates of relative growth rates (daily C-gain). Thus, a system is presented with the ability to answer questions about the nature of in vitro plant physiology related to carbon dynamics, that would otherwise be difficult to assess.

**Abstract:**

Supplemental sugar additives for plant tissue culture cause mixotrophic growth, complicating carbohydrate metabolism and photosynthetic relationships. A unique platform to test and model the photosynthetic proficiency and biomass accumulation of micropropagated plantlets was introduced and applied to *Cannabis sativa* L. (cannabis), an emerging crop with high economic interest. Conventional in vitro systems can hinder the photoautotrophic ability of plantlets due to low light intensity, low vapor pressure deficit, and limited CO_2_ availability. Though exogenous sucrose is routinely added to improve in vitro growth despite reduced photosynthetic capacity, reliance on sugar as a carbon source can also trigger negative responses that are species-dependent. By increasing photosynthetic activity in vitro, these negative consequences can likely be mitigated, facilitating the production of superior specimens with enhanced survivability. The presented methods use an open-flow/force-ventilated gas exchange system and infrared gas analysis to measure the impact of [CO_2_], light, and additional factors on in vitro photosynthesis. This system can be used to answer previously overlooked questions regarding the nature of in vitro plant physiology to enhance plant tissue culture and the overall understanding of in vitro processes, facilitating new research methods and idealized protocols for commercial tissue culture.

## 1. Introduction

Micropropagation allows the rapid and effective propagation and maintenance of uniform plantlets in axenic cultures [1,2,3]. These methods offer value to many emerging industries of high value, such as the developing North American cannabis industry. Unfortunately, the microclimates typically found in micropropagation vessels are characterized by low light intensity, limited CO_2_ availability, and high relative humidity which can impede photoautotrophy and lead to stress-induced ethylene accumulation [4]. Combinations of adverse factors can contribute to culture-induced anatomical aberrations such as hyperhydricity [5], which are commonly associated with cannabis cultures [6]. Ultimately, culture conditions can complicate the development of healthy organs, representing the drawback of maintaining plant materials in vitro [7]. To overcome these setbacks and support in vitro development, sugar is provided as a standardized supplemental carbon supply, enabling photomixotrophic metabolism [8,9]. However, it is generally accepted that sucrose can result in subsequent dependence, which further impedes idealized metabolic functions [10], complicating the acclimation of the plantlets to new environments. Thus, to increase plant quality and ex vitro success, the development of photoautotrophic proficiency is preferred [11,12].

Since the photoautotrophic capability of in vitro specimens is complicated by a cross-talk between light intensity, sugar concentration, and additional atmospheric factors [13], certain questions surrounding the nature of in vitro plant physiology must first be addressed. Though there exists a generalized consensus that rubisco content and activity is affected by sucrose concentration, interpretations are species- and system-specific [14], and the impact of individual and combined factors must further be pursued to develop optimized, species-inclusive protocols. The dynamic equilibria of photoperiodic carbon gains and losses are chief determinants of plant growth and development, such that diurnal CO_2_ fluctuations can be correlated to biomass accumulation [15]. Though CO_2_ produced via dark respiration is rapidly captured during light periods in vitro [16], determining ways to balance gas dynamics in a closed system is challenging, and becomes increasingly problematic when accounting for exogenous carbon sources, such as sucrose, that may sway metabolic processes.

The haze surrounding in vitro photosynthesis can be investigated by integrating light-emitting diode (LED) technology with tissue culture in an open-flow/force-ventilated gas exchange system. Highly manageable in vitro environments that help to investigate plant development and photosynthesis are achievable with these open-flow/force-ventilated photoautotrophic micropropagation strategies. Although these strategies are seldom used for plant tissue culture, it is possible to manipulate and measure the atmospheric properties (such as [CO_2_]) of an in vitro microclimate by relaying a controlled net flow of air through the containment vessel to be gauged by an infrared gas analyzer [17]. Combining this approach with LEDs offers exploratory opportunities that link the synergistic impacts of light quantity, quality, and [CO_2_] to photosynthesis [18,19]. By examining whole-plant transpiration and NCER, their combined influence on whole-plant performance can be determined [20]. Such strategies can be adapted for use with in vitro methods, allowing precise and rapid manipulation of light and [CO_2_] that target physiological responses, in order to address specific questions relating to the nature of in vitro plant development. Thus, a novel platform to investigate the combined influence of abiotic conditions and sucrose on in vitro photosynthesis by controlling sugar, light, humidity, and [CO_2_] in highly replicable settings without extraneous complications was designed. The system was then tested on in vitro *Cannabis sativa* L. (cannabis) as proof of concept.

Systems have previously been devised to quantify the photosynthetic and respiration rates of in vitro plants [21], and studies have revealed that strategic manipulation of light using LEDs allows biomass and secondary metabolite accumulation of in vitro medicinal plants [22,23,24]. Cannabis represents an ideal candidate to model these in vitro growth responses to abiotic factors due to its potential for miniaturization in culture. Additionally, many plants do not readily flower in vitro, but the photoperiodic response of cannabis enables floral development under short-day conditions (unpublished data) and provides potential to study the effects of light quality on floral development and secondary metabolism. Thus, modeling and mapping photosynthetic responses of in vitro cannabis provides the foundation to study light and abiotic influence over a multitude of important physiological characteristics. Ultimately, using this system with cannabis can allow the development of optimized protocols to enhance plant quality and production practices without the need for plant growth regulators, which is of paramount importance for this high-demand, chemical-free industry.

The methods presented allow continuous monitoring of photosynthesis and respiration rates for noninvasive estimation of daily plant growth by quantifying NCER at standardized [CO_2_] [15,25]. Alternatively, destructive sampling can be used after NCER determination to map photosynthetic responses under different conditions, represent data in additional modes, and relate photosynthesis to different parts of the plants [15,19]. These methods can be used to predict growth rates in response to abiotic factors while suggesting optimized treatments to enhance in vitro plant development and photosynthesis. The obtained results can ultimately be used to guide micropropagation system development and precision LED application by modeling the relationships between light intensity, quality, and sugar metabolism in plants that would otherwise be neglected or difficult to conceive.

## 2. Materials and Methods

### 2.1. Plant Material and Culture Conditions

**Figure 1:**
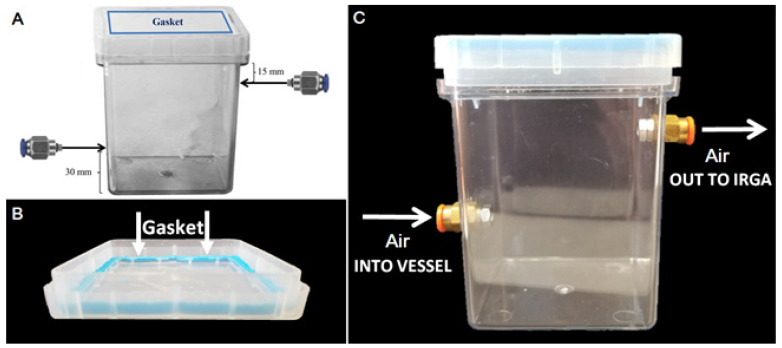
Gas exchange Magenta vessels. Design and functionality of gas exchange culture vessels. (**A**) Culture vessels were modified by fastening nickel-plated brass push-to-connect hose connections for ¼” tubes centered on opposite faces of the GA-7 Magenta vessel, mounted roughly 15 mm from the top lip and 30 mm from the vessel’s bottom. Insertion holes were threaded, and small O-rings were applied to hose connections to allow an air-tight seal. (**B**) To further ensure an air-tight seal for accurate gas exchange measurement, a malleable gasket was applied to the vessels between the lids and edge of box tops by forming “sticky tack” to adhere the two surfaces together. (**C**) Air at a constant flow rate and [CO_2_] was pumped into the bottom hose connection port, allowing gas to fill the vessel from the bottom, and then relayed to the IRGA from the upper hose connection port.

**Figure 2:**
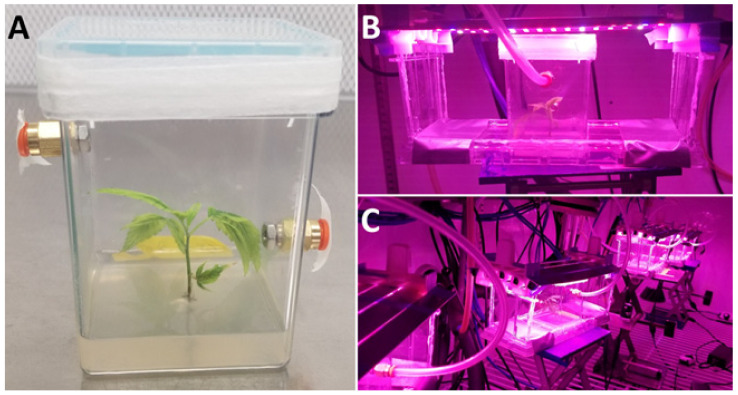
Cultured plantlets connected to the open-flow/force-ventilated gas exchange system. (**A**) Experimental culture for experiment 1 removed from acclimation for experimentation. (**B**) Experimental culture connected to the open-flow/force-ventilated gas exchange system with light lids suspended above the culture vessel. (**C**) Experimental cultures connected to the open-flow/force-ventilated gas exchange system, with light lids suspended above cultures during experimentation.

For these experiments, cultures of *Cannabis sativa,* accession RTG-XX were used (see Monthony et al. [26], for SNP-based genetic fingerprint). The plantlets were well established and had been maintained over long periods with regular subculturing occurring at 4–6 week intervals prior to this experiment. The cultures were stored at 27 ℃, under an 18 h photoperiod, 50 µmol m^−2^ s^−1^ PPFD (photosynthetic photon flux density), comprised of 75% R (600–700 nm), 12.5% B (400–500 nm), and 12.5% W (400–700 nm) for several weeks before experimentation. Multinodal explants with 5–7 nodes inclusive of apical meristems were excised from parental cultures and then subcultured to identical media containing 0.1% (*v*/*v*) antimicrobial plant preservation mixture, 0.532% (*w*/*v*) DKW, and 0.6% (*w*/*v*) agar, with pH adjusted to 5.7 before agar addition and sterilization. Medium components were obtained from PhytoTech Labs, Inc. (Lenexa, KS, USA). For experiment 1, explants were cultured with 3% (*w*/*v*) sucrose and no growth regulators. For experiment 2, explants were cultured without sucrose and with 2.5 µM IBA. Experimental cultures were maintained in customized Magenta vessels (GA-7, Magenta LLC, Lockport, IL, USA) (Figure 1 and Figure 2A). These customized Magenta vessels enabled gas exchange measurement during the experiments.

### 2.2. Gas Exchange System

Details of the experimental gas exchange system were similar to those described by Leonardos and Grodzinski [27] and Lanoue, Leonardos, and Grodzinski [18]. We describe the applied methods in reference to their publication as follows:

Each round of experimentation included four Magenta vessels with explants (experimental) and a negative control with only medium, which were connected to an open-flow/force-ventilated gas exchange system (Figure 2C). These five vessels were kept inside a plant growth chamber (Big Foot GS20 BDAF LT, Biochambers, MB, Canada), in which the experiments took place. Light (from custom LEDs), relative humidity, temperature, and CO_2_ were maintained at consistent levels. Compressed air traversed a FT-IR purge generator (model 75–53, Parker Balston Analytical Gas Systems, Haverhill, MA, USA) to remove H_2_O and CO_2_ before being integrated with pure CO_2_ from a cylinder using two mass flow controllers (Smart-Trak 100 series, Sierra Instruments Inc., Monterey, CA, USA) to achieve the [CO_2_] of interest. For the experiments presented here, the system delivered calibrated concentrations of ambient (400 ppm) or elevated (1200 ppm) CO_2_. Another mass flow controller could be used to achieve different [O_2_] values if required by mixing appropriate ratios of N_2_, CO_2_, and O_2_ gases.

Additional experimental conditions in each vessel included temperatures of 26 °C and relative humidity > 85%. The humidity of the gas entering the vessels was maintained by streaming the implemented volume of gas from the two, previously described, mass flow controllers through a gas bubbler held in a water bath (RTE17 NESLAB, Thermo Fisher Scientific, Waltham, MA, USA) at a constant temperature equal to the desired dew point inside the Magenta experimental vessels. Gas lines were outfitted through heating cables, with temperatures held consistently above the dew point to prevent condensation. Calibrated flow rates of air were then split between the four experimental vessels using four mass flow controllers (Smart-Trak 100 series, Sierra Instruments Inc., Monterey, CA, USA) and the control/reference vessel, which was controlled using a variable rate flow meter (model N112-02, Cole-Parmer Instruments Co., Vernon Hills, IL, USA) which also allowed additional control over air pressure entering the mass flow controllers. The flow rate of air through each experimental vessel was maintained at 100 cc min^−1^. An electronic flow meter (Smart-Trak series 100, Sierra Instruments Inc., Monterey, CA, USA) quantified the air emerging from the vessels, allowing us to detect vessel leaks by comparing flow rates into and out of the vessels. A series of manifold solenoid valves (Model 8320G222/3 Red-Hat, ASCO, Alexandria, NJ, USA) directed the gas emerging from each vessel line, one at a time, to an infrared gas analyzer (IRGA) (model 7000 CO_2_/H_2_O, Li-Cor, Biosciences, Lincoln, NE, USA).

A specialized integration of electronic hardware and software (LabView 2017, National Instruments Corporation, Austin, TX, USA) installed on a personal computer (690 Precision, Dell, Round Rock, TX, USA) allowed monitoring, logging, and control of all integrated devices used in the system. The custom-made system software allowed manual and automatic control and monitoring of all instruments and environmental parameters including the IRGA and the [CO_2_] in each experimental vessel and the reference vessel.

### 2.3. Experimental Lighting

**Figure 3:**
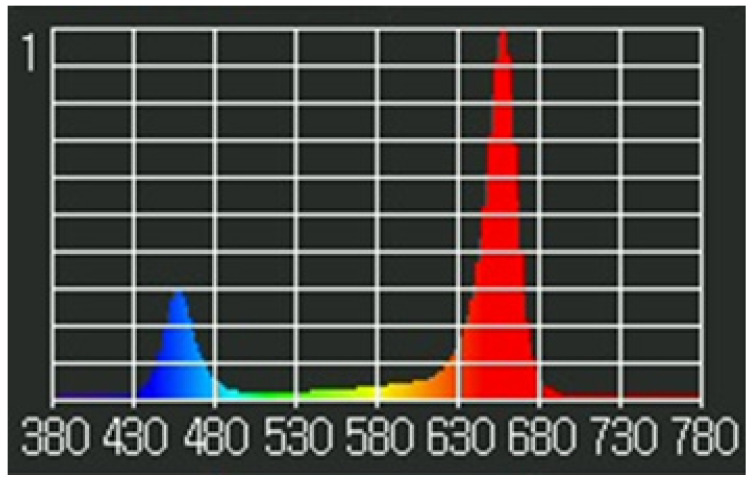
Spectral analysis of the LED lights. Relative spectrum of light emitted by the LEDs that were implemented throughout experimentation. LEDs used had peak wavelengths of blue (456 nm) and red (657 nm), with some white LEDs for a broad spectrum. Spectral analysis was obtained using a Li-Cor LI-180.

LEDs were used as the light source for the conducted experiments. These light sources allowed minimal heat dissipation and offered the potential to alter irradiance in a direct and precise manner. Light lids similar to those described by Fang et al. [28] and Shukla et al. [29] emitted 75% R (600–700 nm), 12.5% B (400–500 nm), and 12.5% W (400–700 nm) (Figure 3), as previously described. Rapid and precise manipulation of light intensity was facilitated by connecting LED to PWM-based dimming switches, offering a gradient of light intensities to track photosynthetic responses using photosynthetic light response curves. Experimental light intensities included 0, 25, 50, 75, 100, 150, 250, and 400 µmol m^−2^ s^−1^ PPFD for experiment 1. For experiment 2, light intensity was maintained at 50 µmol m^−2^ s^−1^ PPFD. Light lids were suspended above culture vessels at uniform heights using mounts (Figure 2B). Experimental irradiance levels were pre-calibrated at plantlet height from within a modified empty Magenta vessel using a spectrometer (Li-Cor LI-180, Li-Cor Biosciences, Lincoln, NE, USA).

### 2.4. Experimental Process 1

Apical explants were subcultured from parent cultures into customized gas exchange Magenta vessels containing identical media as parent cultures (previously described). These new cultures were set to acclimate in a growth chamber for 24–72 h, with identical conditions as the parental cultures (previously described). The experiment was conducted over two rounds, using a total of eight plantlets (*n* = 8). Each experimental round spanned 2 days. On day 1 of experimentation, we tested [CO_2_] at 400 ppm. Four experimental cultures (acclimated for 24 h) and one control/reference vessel (empty, with medium alone) were connected to the gas exchange system. Once it was determined that there were no air leaks, cultures remained in darkness until a steady-state respiration rate was achieved after roughly 30–40 min. Lights were then illuminated to the intensity to which plantlets were acclimated (50 µmol m^−2^ s^−1^). This intensity was maintained for approximately 30–40 min until a steady state of photosynthesis was achieved. Irradiance was then augmented to the maximum experimental intensity of 400 µmol m^−2^ s^−1^, and then sequentially reduced to, 250, 150, 100, 75, 50, 25, and 0 µmol m^−2^ s^−1^. Each experimental light intensity was maintained for a minimum of 25 min, until steady-state [CO_2_] on the IRGA was observed. The following day, this process was repeated with the same set of four experimental cultures and one control/reference unit at elevated [CO_2_] (1200 ppm). On day 3 of experimentation (day 1 or round 2), four new experimental cultures (acclimated for 72 h) and one new control/reference unit were used in the above protocol. To normalize the data, round 2 of experimentation started with elevated [CO_2_] (1200 ppm) on day 3 of experimentation (day 1 of round 2), followed by ambient [CO_2_] (400 ppm) on day 4 of the experiment (day 2 of round 2). Data from each CO_2_ treatment repetition were pooled together to negate any errors due to order of treatments.

### 2.5. Experimental Process 2

**Figure 4:**
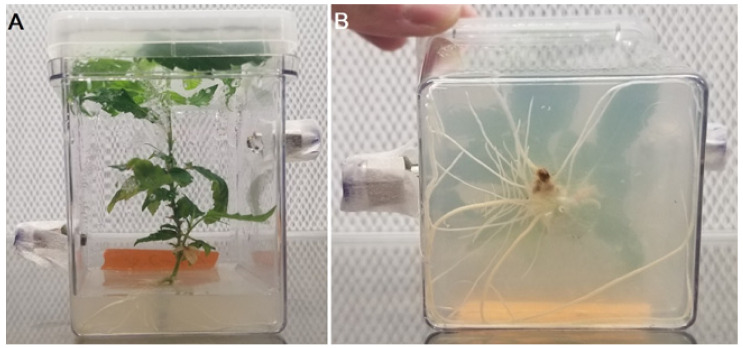
Experimental culture removed from acclimation for experiment 2. (**A**) Depicting above-ground biomass when removed from acclimation before the experiment (**B**) Showing root development of experimental specimen, depicting below-ground biomass.

Apical explants were subcultured from parent cultures into customized gas exchange Magenta vessels containing medium supplemented with 2.5 µM IBA and no sucrose. All other medium components were identical to parent cultures (previously described). Experimental cultures (Figure 4A) were maintained in identical conditions to parental cultures (previously described) for roughly 7 weeks until appreciable roots developed (Figure 4B). This experiment was completed over one 24 h period with four plants (*n* = 4). A single control/reference gas exchange Magenta vessel and the four experimental cultures were connected to the gas exchange system. The vessels were checked for leaks, and lights were powered on to 50 µmol m^−2^ s^−1^ PPFD. Lights remained illuminated at 50 µmol m^−2^ s^−1^ PPFD for an 18 h period with continuous NCER measurements being recorded. After 18 h, the lights were turned off and cultures remained in darkness for 6 h. Continuous NCER measurements were recorded during the dark period, in the same way as during the light period.

### 2.6. Data Harvesting and Processing

After each round of experimentation, leaf and stem fresh weights were collected and leaf surface areas were quantified using ImageJ software [30]. Leaf and stem dry weights were obtained after heating samples in a convection oven at roughly 80 °C for 48 h.

Net carbon exchange rates (NCERs) of mixotrophic *Cannabis* cultures on a per leaf area basis were calculated using Equation (1):

NCER = (Flow Rate × Density _CO2_ × ([CO_2_] _Reference_ − [CO_2_] _Sample_))/Leaf Area (1)

where NCER is in µmol CO_2_ m^−2^ s^−1^; Flow Rate is the gas flow rate through each vessel; Density _CO2_ is the density of CO_2_ at the experimental temperature; [CO_2_] _Reference_ and [CO_2_] _Sample_ are the [CO_2_] of the reference and sample vessel detected by the IRGA; and Leaf Area is the total leaf area of the plantlet.

NCER can also be expressed on different bases depending on the objectives of an experiment. For example, it can be expressed on a per plantlet, chlorophyll, or dry mass basis.

Net carbon gain (C-gain) throughout the experimental period was calculated by summation of carbon gains during the light and carbon losses during dark periods using Equation (2):

C-gain = ∑(NCER _Daytime_ × t_1_) + ∑(NCER _Nighttime_ × t_2_) (2)

where C-gain is in mg C m^−2^; NCER _Daytime_ is the positive NCER due to photosynthesis in the light and NCER _Nighttime_ is the negative NCER due to respiration in the dark; and t_1_ and t_2_ are the time of the light and dark periods, respectively.

C-gain can also be expressed on a per plantlet or dry mass basis. By measuring the C content of the plantlets, one can also calculate relative growth rates, which are traditionally expressed as dry mass gained per dry mass present per unit of time. Thus, this method allows for estimation of relative growth rates over short periods (e.g., a day) using a small number of plantlets, which would not be possible by employing traditional destructive methods of measuring relative growth rates that require a large number of samples to be harvested over larger periods of time (e.g., weeks, months).

Light–photosynthetic response curves were created using a three-parameter exponential rise to a maximum nonlinear function (Equation (3)) in SigmaPlot 10 (Systat Software Inc., San Jose, CA, USA) to fit the NCER response of the plantlets to the different PPFD levels:

NCER = y_o_ + a (1 − e^(−bx)^) (3)

where NCER is in µmol CO_2_ m^−2^ s^−1^; y_o_ is the NCER at 0 μmol m^−2^ s^−1^ PPFD; x is the PPFD; e is a mathematical constant approximately equal to 2.71828; and a, b are coefficients.

## 3. Results

### 3.1. Experiment 1

**Figure 5:**
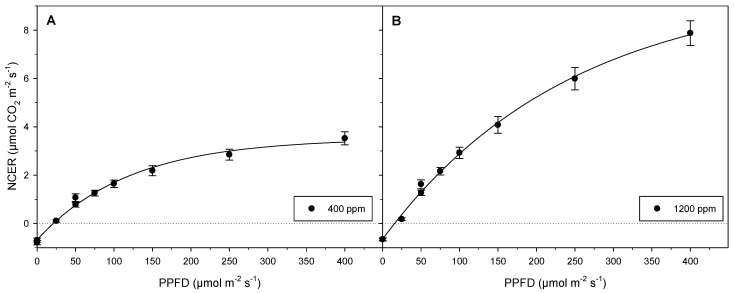
Light–photosynthetic response curves based on mean NCERs from experiment 1. Data represent average NCERs of 8 plantlets (*n* = 8). Error bars represent standard errors of mean. Depicted are (**A**) NCER values at 400 ppm [CO_2_] (**B**) NCER values at 1200 ppm normalized by leaf areas. Two mean values at 0 µmol m^−2^ s^−1^ PPFD are indicative of respiration prior to and following light exposure, respectively. Two means at 50 µmol m^−2^ s^−1^ PPFD show NCERs during initial exposure from darkness to 50 µmol m^−2^ s^−1^ PPFD and exposure to the same light intensity when sequentially reduced from 400 µmol m^−2^ s^−1^ PPFD back to darkness. Respiration rates were similar before and after the light exposures, whereas NCERs at 50 µmol m^−2^ s^−1^ PPFD were reduced by approximately 10–20% at the end of experimentation.

Integrating an open-flow/force-ventilated gas exchange analysis system with customized gas exchange micropropagation vessels and dimmable LED light lids allowed calculation of the photosynthetic capacity of mixotrophic cannabis explants (Figure 5) influenced by a combination of different exogenous conditions (light, [CO_2_], sucrose, relative humidity). With 3% (*w*/*v*) sucrose, the photosynthetic capacity at different light intensities and [CO_2_] of the tested cannabis cultures was effectively determined with high accuracy, in a relatively short timeframe. The results clearly show that this high-throughput method can be used to produce large datasets based on NCER for multiple analyses relating to CO_2_ assimilation, transpiration, and photosynthetic responses.

The NCERs of unrooted, mixotrophic cannabis cultures provided with consistent [CO_2_] increased with increasing PPFD. This trend was further amplified when [CO_2_] was augmented (Figure 5). At each [CO_2_] value, respiration rates before and after light exposure showed comparable values (Figure 5). Light compensation occurred at higher light intensity for the lower tested [CO_2_] values (400 ppm), while at the higher tested [CO_2_] values (1200 ppm), light compensation occurred at a lower light intensity (Figure 5). NCERs at 50 µmol m^−2^ s^−1^ PPFD, a common in vitro light condition [31], were reduced by roughly 10–20% toward the end of experimentation compared to at the beginning for both [CO_2_] values tested (Figure 5). NCER was shown to approach a plateau at 400 µmol m^−2^ s^−1^ PPFD with [CO_2_] maintained at 400 ppm, while NCER remained on the ascent at 400 µmol m^−2^ s^−1^ PPFD at 1200 ppm [CO_2_] (Figure 5). Full light saturation was nearly achieved at low [CO_2_] (Figure 5A), but clearly not achieved at high [CO_2_]. (Figure 5B).

### 3.2. Experiment 2

**Figure 6:**
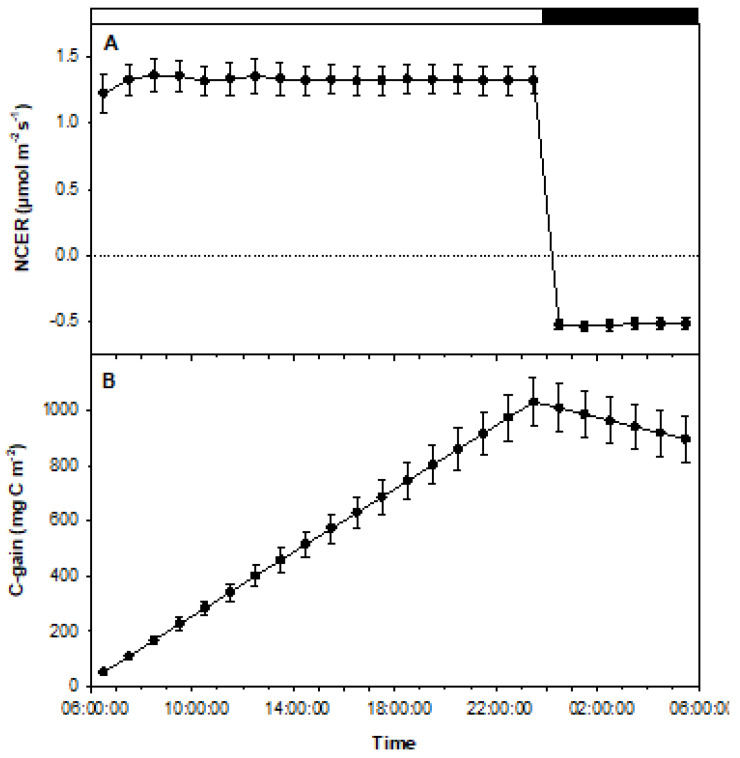
Graphical representations of the data collected from experiment 2. Data represent average NCERs of 4 fully rooted plantlets (*n* = 4). (**A**) Positive values indicate photosynthetic rates during the period of illumination, while negative values represent respiration during the period of darkness. Error bars signify mean standard errors. (**B**) Tracking carbon gains and losses throughout the experiment based on NCER values during light and dark periods. C-gains increased during light periods and decreased in darkness. Error bars represent mean standard errors.

The open-flow/force-ventilated gas exchange micropropropagation system combined with calibrated LED light lids allowed diurnal assessment of photosynthesis and respiration, enabling estimation of net carbon gain (C-gain) (Figure 6). This permitted nondestructive tracking and forecasting of biomass accumulation as a function of combined photosynthetic and respiration rates in response to different environmental factors (light quality/intensity, media components, gaseous environment, etc.). The results indicate that composite datasets can be created to relate the importance of various individual, combinational, or synergistic influences to growth rate and duration of culture periods.

For these sucrose-free, rooted cultures, the results clearly illustrate that NCER remained consistent when [CO_2_] was maintained at 400 ppm and light intensity remained at 50 µmol m^−2^ s^−1^ PPFD (Figure 6A). Also shown is the continuously stable negative NCER during the dark period (Figure 6A). There was a slight increase in NCER at the beginning of the experiment as lights were turned on, before stabilization was achieved. When lights were turned off, a quick drop in NCER and rapid stabilization was observed at a level indicative of respiration (Figure 6A). Also shown is a stable rise of C-gain during light periods, with a steady decrease of C-gain throughout the period of darkness (Figure 6B).

## 4. Discussion

While other systems devised to measure in vitro photosynthesis rely on air exchange estimates between the culture vessel and external environment [32,33], the method described here more accurately measures in vitro gas usage to better evaluate physiological implications. The presented data clearly convey that evaluation of NCER in vitro becomes a high-precision, swift, and accurate task when irradiance and [CO_2_] can be precisely adjusted using the developed system. The open-flow/force-ventilated micropropagation method presented, together with LEDs, allows high control over and adjustment of substrate, atmospheric conditions, and light influences which are more difficult to achieve and maintain in greenhouses and other controlled environment settings. Using these methods along with the platform presented, it is possible to clarify preconceived notions about the nature of in vitro plant responses. Comprehensive experiments with high statistical validity can be carried out in a matter of days to progress research initiatives and optimize protocols for a wide range of applications. Furthermore, in vitro cannabis production allows the elimination of biotic contamination factors while contributing genetically and physiologically uniform, miniaturized specimens to improve replicability. Although certain plants do not flower in vitro, many cannabis cultivars develop floral organs under short-day conditions [34,35], which is also observed in vitro [36], further extending the applicability of these protocols with cannabis to assess additional light-mediated physiological responses and secondary metabolism. With expected trends emerging from both experiments, assessing and validating specific suppositions relating to the nature of plant responses as directed by different growth conditions using the methods presented exemplifies their value. NCER and C-gain data can be expressed in different ways, including on per plantlet, leaf area, chlorophyll, or dry weight bases. For example, photosynthesis rates have traditionally been expressed per leaf area or chlorophyll, whereas relative growth rates are usually presented in mass gained per total mass over time. If either NCER or C-gain are expressed on a per plantlet basis, they are truly nondestructive estimates and allow for repeated measurements over time. The light–response curves obtained based on NCER at different [CO_2_] values and light over a short period verified the effectiveness of this system to gauge and model in vitro photosynthesis. Efficacy was further verified when applied over a long term to quantify NCER and C-gain at consistent [CO_2_] and irradiance levels throughout a diurnal experiment.

For experiment 1, it was hypothesized that lack of photosynthetic light saturation would not be due to low light intensities, but the result of mutual shading of the leaves within the canopy or additional factors. For ex vitro plants, the mutual shading effect causes a reduction in intercepted irradiance [37] that increases toward the basal canopy [38]. The influence of PPFD on canopy light saturation of micropropagated specimens, though at lower light intensities in vitro, would be analogous to mutual canopy shading in ex vitro plants grown in controlled environments. Pepe et al. [39] indicated that cannabis functions well at higher light intensities than those normally used for micropropagation. In the current study, since normal in vitro irradiance levels were raised by a factor of 8 and plantlets neared photosynthetic saturation at low [CO_2_] but remained far from saturation at high [CO_2_], it is expected that light saturation was limited by factors other than the irradiance levels provided. Rodriguez-Morrison et al. [40] found that dry mass flower yield from ex vitro cannabis harvest index increased linearly up to 1800 µmol m^−2^ s^−1^, although saturating photosynthetic light intensities were achieved well under 1800 µmol m^−2^ s^−1^ at 400 ppm [CO_2_]. Chandra et al. [41] found that net photosynthesis of four medicinal varieties of cannabis increased at elevated [CO_2_] with irradiance maintained at 1500 µmol m^−2^ s^−1^ when CO_2_ was supplied over 1000 ppm. Even though the combined effects of supplemental CO_2_ and high irradiance can enhance ex vitro photosynthesis [42,43], maintaining [CO_2_] at 400 ppm would have been appreciable for photosynthesis, since in vitro [CO_2_] regularly fluctuates below this value [16]. Correspondingly, increasing PPFD can further optimize photosynthetic growth with CO_2_ fertilization [44]. Augmenting light intensity and [CO_2_] positively impacts growth and improves quality of both *Doritaenopsis* [45] and *Theobroma cacao* [46] when grown in vitro. These findings are in accordance with the obtained results, since the NCERs achieved with 125 µmol m^−2^ s^−1^ PPFD and 1200 ppm [CO_2_] were comparable to those attained at 400 µmol m^−2^ s^−1^ PPFD with 400 ppm [CO_2_], indicating the potential for more efficient use of light when [CO_2_] is not limited. Though formal assessments of physiological responses to abiotic conditions are beyond the scope of this paper, the results obtained support the idea that photosynthetic constraint in vitro could be due to low CO_2_ availability [47], and counters the common view that sucrose negatively impacts photosynthesis [48], at least for cannabis. Ultimately, the methods presented in this paper can be used in future experiments that focus on in vitro plant physiology to evaluate the impacts of different medium components such as sucrose and plant growth regulators in addition to [CO_2_] and light to determine their influences on photosynthesis.

When comparing photosynthetic activity at the beginning and end of experiment 1 (at 50 µmol m^−2^ s^−1^ PPFD and in darkness), a 10–20% reduction in NCER was observed toward the end of the experiment at 50 µmol m^−2^ s^−1^ PPFD, and almost no change in dark NCER (Figure 5). One possible explanation for the decrease in NCER after performing the light–response curves is the engagement of nonphotochemical quenching, considering the in vitro plantlets were acclimated to low light intensity (50 µmol m^−2^ s^−1^ PPFD). Although assessment is beyond the scope of this methods paper, this result does show the power of the system to intricately map photosynthetic performance at different light intensities and to match light intensities within a span of time. This ability will ultimately allow further investigations leading to a deeper understanding of certain biological mechanisms related to these findings. In the future, experiment 1 can also be adapted to compare NCERs with various plant growth regulators and sucrose concentrations in media, in addition to different light intensities and [CO_2_] values. This will eventually help to identify limiting factors relating to photosynthetic performance and allow precise treatment modifications to improve photosynthesis. The impact of different influential factors on respiration can also be assessed. When alternative treatments are compared to controls that simulate conventional tissue culture methods, the impact of different combinations of factors on in vitro photosynthetic capacity can be evaluated.

Experiment 2 also showed that nondestructive tracking of in vitro photosynthesis and respiration in response to specific growth conditions over prolonged periods is achievable with high precision. This is important when considering the applicability of new methods for long-term culture maintenance and utility throughout different culture growth phases. In conventional tissue culture systems, [CO_2_] fluctuations are triggered by photosynthetic and respiratory responses between light and dark intervals, whereby CO_2_ evolution during dark respiration is rapidly assimilated during periods of illumination [16]. Light period assimilation results in diminishing [CO_2_], which is likely reduced to the CO_2_ compensation point. Negative environmental impacts can be mitigated by integrating 3% (*w*/*v*) sucrose into the growth media, which is commonly added to allow mixotrophic metabolism to emerge [39,48]. However, this strategy can have direct species-specific implications for photosynthetic performance [49], medium water potential [50], and undoubtably, C-gain. Experiment 2 showed that maintaining consistent [CO_2_] and irradiance for sucrose-free cultures can allow NCER to stabilize over prolonged periods of time. Such trends directly relate photosynthesis and respiration to cumulative C-gains and losses, which highlights the ability to track metabolic processes relative to biomass accumulation and culture conditions using the methods presented. It is thus practical that the developed system can be used to enhance in vitro protocols to allow long-term maintenance of photosynthetic, sucrose-free cultures and limit culture-induced abnormalities while enhancing ex vitro adaptability. Future experiments using the methods applied in experiment 2 should investigate differences in photosynthesis and C-gain throughout short-day and long-day photoperiods and test different equations to process and represent the data obtained (per plant, per leaf, per dry mass, per chlorophyll, etc.) [19] for enhanced accuracy. Additionally, different light intensities, light qualities, sucrose levels, and [CO_2_] values should be examined to gauge NCER stability and differences in C-gain in response to various conditions over alternative time intervals.

By modeling in vitro performance, the platform described makes it conceivable to develop basic hypotheses about certain physiological responses of cultured plants to abiotic influences, and perhaps even to apply those deductions to ex vitro plants, allowing time and space to be saved during preliminary trials. The results show the accuracy of this technique to precisely map plant performance under different environmental conditions and with different medium additives. This will enable more comprehensive investigations to examine different physiological mechanisms associated with certain plant responses. Using this system to assess numerous growth dynamics and answer complex questions about specific in vitro plant responses throughout a set growth period will allow model development and enhanced precision in prospecting for plant tissue culture methods.

## 5. Conclusions

Though follow-up experiments must be completed to further illuminate the vista of in vitro cannabis photosynthesis, these results confirm the validity of this system for precisely measuring in vitro gas exchange in relation to photosynthetic capacity and C-gain. This powerful system can be used to assess currently held assumptions and answer previously overlooked questions, offering further insight to mixotrophic and/or photoautotrophic performance of plantlets in culture. Although in vitro irradiance is generally kept low (~50 µmol m^−2^ s^−1^) [16,51], this paper demonstrates that improved abiotic conditions for enhanced in vitro performance, and perhaps better ex vitro adaptability, can be identified by changing abiotic factors and quantifying plant responses. Additionally, this platform can be used to measure photosynthesis over long periods in a manner that allows C-gains to be quantified based on photosynthetic capacity in a non-destructive manner. The methods described allow us to test the efficacy of current protocols, in addition to prospecting alternative procedures for enhanced in vitro performance. Ultimately, optimizing in vitro performance by enhancing abiotic conditions can dissuade the emergence of culture-induced phenotypes. Additionally, miniaturized cannabis is ideal for studying in vitro photosynthetic responses. Using LEDs in conjunction with accurate gas exchange measurements improves the speed, accuracy, and replicability of experimental procedures that can advance the current understanding of photosynthesis. These protocols can also be combined with supplementary methods relating to machine learning, metabolomics, and genomics to more accurately model co-active relationships between light, CO_2_, and sucrose metabolism to optimize plant development evaluations. Using the presented system to model and prospect different physiological responses to influential factors will ultimately enhance the precision of plant tissue culture methods.

## Data Availability

Not applicable.
